# Rituximab for nephrotic syndrome in children

**DOI:** 10.1007/s10157-016-1313-5

**Published:** 2016-07-15

**Authors:** Kazumoto Iijima, Mayumi Sako, Kandai Nozu

**Affiliations:** 1 Department of Pediatrics, Kobe University Graduate School of Medicine, 7-5-2 Kusunoki-cho, Chuo-ku, Kobe, 650-0017 Japan; 2 Division for Clinical Trials, Department of Clinical Research, Center for Clinical Research and Development, National Center for Child Health and Development, 2-10-1 Okura, Setagaya-ku, Tokyo, 157-8535 Japan

**Keywords:** Idiopathic nephrotic syndrome, Complicated frequently relapsing/steroid-dependent nephrotic syndrome, Rituximab, Proteinuria, Children

## Abstract

Idiopathic nephrotic syndrome is the most common chronic glomerular disease in children. At least 20 % of children with this syndrome show frequent relapses and/or steroid dependence during or after immunosuppressive therapies, a condition defined as complicated frequently relapsing/steroid-dependent nephrotic syndrome (FRNS/SDNS). Approximately 1–3 % of children with idiopathic nephrotic syndrome are resistant to steroids and all immunosuppressive agents, a condition defined as refractory steroid-resistant nephrotic syndrome (SRNS); these SRNS children have a high risk of end-stage renal failure. Rituximab, a chimeric anti-CD20 monoclonal antibody, has been shown to be effective for patients with complicated FRNS/SDNS and refractory SRNS. This review describes the recent results of rituximab treatment applied to pediatric nephrotic syndrome, as well as those of our recent study, a multicenter, double-blind, randomized, placebo-controlled trial of rituximab for childhood-onset complicated FRNS/SDNS (RCRNS01). The overall efficacy and safety of rituximab for this disease are discussed.

## Introduction

Idiopathic nephrotic syndrome is the most common chronic glomerular disease in children, occurring in two of 100,000 children per year in Western countries [1] and in five of 100,000 children per year in Japan. Approximately 80 % of these children have minimal change nephrotic syndrome, most of whom respond well to steroid therapy, steroid-sensitive nephrotic syndrome (SSNS) [2]. However, up to 50 % of these SSNS patients, develop frequently relapsing nephrotic syndrome (FRNS), which is defined as at least four relapses per year or at least two within 6 months of the initial presentation. Conversely, these SSNS patients may develop steroid-dependent nephrotic syndrome (SDNS), defined as two consecutive relapses during tapering or within 14 days of cessation of steroid therapy. Fifty to sixty percent of children with FRNS meet definition of SDNS. These definitions are from the International Study of Kidney Disease in Children (ISKDC) criteria [3]. In addition, 10–20 % of patients with idiopathic nephrotic syndrome have steroid-resistant nephrotic syndrome (SRNS), defined as persistent proteinuria after a 4- to 8-week course of oral prednisolone [3].

**Fig. 1:**
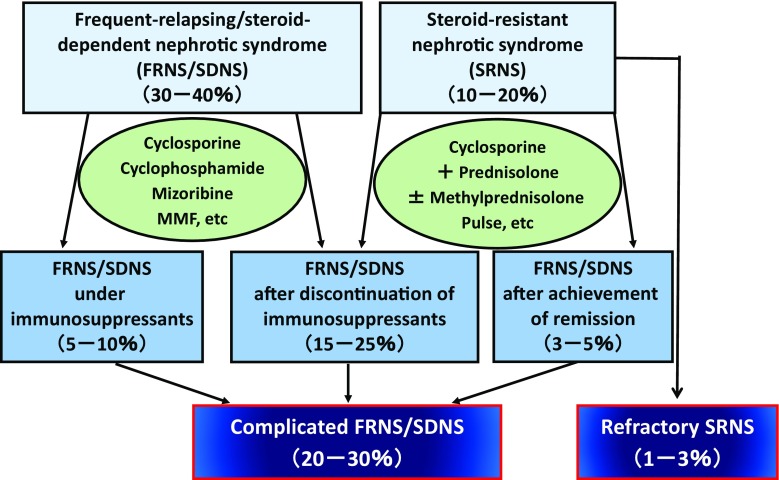
Complicated frequent-relapsing/steroid-dependent nephrotic syndrome and steroid-refractory nephrotic syndrome. *MMF* mycophenolate mofetil

Standard treatments worldwide for FRNS/SDNS in children are immunosuppressive agents, including cyclophosphamide, chlorambucil, cyclosporine (CyA), tacrolimus, and levamisole [4], whereas the standard treatment for SRNS in children is CyA [5]. The 2013 Clinical Practice Guidelines for Pediatric Nephrotic Syndrome of the Japanese Society for Pediatric Nephrology recommend CyA, cyclophosphamide or mizoribine as drug therapy for children with FRNS/SDNS, and CyA for children with SRNS [6]. Although these treatments are generally successful in most patients, some endure a complicated clinical course; 10–20 % of children with FRNS/SDNS receiving CyA showed frequent relapses [7, 8], and approximately 30 % of children with SRNS had frequent, steroid-sensitive relapses after complete remission [9]. In addition to a lack of efficacy in some patients, CyA can induce side effects, in particular chronic nephrotoxicity [10], suggesting that after the long-term use, CyA should be discontinued. However, CyA discontinuation generally results in frequent relapses or steroid dependence, requiring long-term steroid treatment, posing a long-term risk to children. Collectively, at least 20 % of children with idiopathic nephrotic syndrome show frequent relapses or steroid dependence during or after immunosuppressive therapies, a condition defined as “complicated FRNS/SDNS”. Additionally, approximately 1–3 % of children with idiopathic nephrotic syndrome show resistance to steroids and immunosuppressive agents, putting them at high risk of end-stage renal failure, a condition defined as “refractory SRNS” (Fig. 1). The failure of current therapies suggests the need for new agents to treat complicated FRNS/SDNS and refractory SRNS.

Rituximab, a chimeric anti-CD20 monoclonal antibody originally developed to treat patients with B cell non-Hodgkin’s lymphoma, is now used in the treatment of various autoimmune diseases, such as Wegener’s granulomatosis, rheumatoid arthritis, and microscopic polyangiitis. Many studies in the past decade have reported the effectiveness of rituximab for complicated FRNS/SDNS [11] and refractory SRNS [12] as defined in Table 1. In this review we describe studies on the use of rituximab to treat nephrotic syndrome in children, including our recent work, updating our previous review [13] with postmarketing results, and discuss the efficacy and safety of rituximab in patients with this disease.

**Table 1:** Definitions of complicated FRNS/SDNS and refractory SRNS

Term	Definition
Complicated FRNS/SDNS	Patients were diagnosed with complicated FRNS/SDNS if they showed one of the following: 1. frequent relapses or steroid dependence after completion of treatment with immunosuppressive agents, such as cyclosporine, cyclophosphamide, mizoribine, or mycophenolate mofetil 2. frequent relapse or steroid dependence during immunosuppressive drug therapy 3. a history of steroid resistance, with frequent relapse or steroid dependence during or after the completion of immunosuppressive drug therapy [11]
Refractory FRNS	Patients were diagnosed with refractory SRNS when the combination of steroids and immunosuppressive agents including calcineurin inhibitors did not lead to remission [12]

*FRNS* frequently relapsing nephrotic syndrome, *SDNS* steroid-dependent nephrotic syndrome, *SRNS* steroid-resistant nephrotic syndrome

## Mechanisms of action of rituximab

The pathogenesis of nephrotic syndrome remains uncertain. More than 40 years ago, nephrotic syndrome was hypothesized to be primarily a disorder of T cell function [14]. B cells induce T cell activation, mediate antibody-independent autoimmune damage, and express costimulatory molecules and cytokines, maintaining T cell activation in autoimmune diseases. Rituximab treatment leads to B cell depletion caused by B cell apoptosis, antibody-dependent cellular cytotoxicity or phagocytosis, suppressing interactions between B cells and T cells, which may prevent relapses in patients with nephrotic syndrome. Regulatory T cell (Treg) function has been reported to be impaired in patients with minimal change nephrotic syndrome, and Treg cells have been found to induce remission in nephrotic syndrome [15, 16]. Rituximab may therefore enhance the number and function of Treg cells [17], suggesting that rituximab maintenance of remission in patients with nephrotic syndrome is due to the restoration of Treg cell function. However, nephrotic syndrome may actually be caused by B cell-derived factors, including B cell cytokines and autoantibodies.

Acid sphingomyelinase-like phosphodiesterase 3b (SMPDL-3b) plays a role in the conversion of sphingomyelin to ceramide by acid sphingomyelinase (ASMase) and its levels are reduced in renal biopsy specimens from patients with recurrent focal segmental glomerulosclerosis (FSGS). Moreover, decreased SMPDL-3b expression is associated with an increased susceptibility of podocytes to injury after exposure to sera from these patients. Rituximab has been reported to bind directly to SMPDL-3b on the cell surface of podocytes, modulate the activity of ASMase and regulate the generation of ceramide, thereby stabilizing podocyte structure and function and preventing recurrent FSGS [18]. Further studies are needed to clarify whether rituximab has similar mechanisms of action in complicated FRNS/SDNS and refractory SRNS.

## Rituximab treatment for recurrent nephrotic syndrome after renal transplantation

Rituximab treatment for patients with recurrent nephrotic syndrome and posttransplant lymphoproliferative disorder (PTLD) after renal transplantation was shown to induce long-term remission of both nephrotic syndrome and PTLD [19]. In contrast, rituximab failed to improve nephrotic syndrome in renal transplant patients with recurrent FSGS [20]. Members of the International Pediatric Nephrology Association were asked to retrospectively complete a questionnaire describing the use of rituximab in their center; in that survey 60 % of patients with post-transplant recurrence of nephrotic syndrome had a good initial response to rituximab [21]. A response was seen in 81 % of the pediatric cases reported in the literature as compared to 50 % of adult patients [22]. A systematic review revealed that a younger age at transplant and normal serum albumin level at recurrence may predict response [23].

## Rituximab treatment for refractory steroid-resistant nephrotic syndrome

Bagga et al. reported, for the first time, that rituximab was effective for refractory SRNS. In this report, rituximab treatment of five children with refractory SRNS induced complete remission in three patients and partial remission in two [24]. Additionally, rituximab induced complete remission in two children with refractory SRNS and FSGS [25]. These findings, as well as other reported cases, suggested that rituximab as an effective therapy for some patients with refractory SRNS [21, 26, 27]. An open-label, randomized trial of 31 children with refractory SRNS compared responses in 16 children who received calcineurin inhibitors, prednisolone, and two infusions of rituximab, and in 15 who received calcineurin inhibitors and prednisolone alone [28]. However, proteinuria remained unchanged in rituximab-treated patients and none achieved partial or complete remission. Thus, to date no evidence is available for rituximab as an effective therapy for patients with refractory SRNS. Whether the histological subtype has any influence on the response to rituximab is controversial. Sinha et al. reported that FSGS is associated with higher odds of non-response [29], whereas Magnasco et al. showed that no factors including histologic findings affect the outcome [28].

## Rituximab treatment for complicated frequently relapsing nephrotic syndrome/steroid-dependent nephrotic syndrome

Benz et al. reported, for the first time, the efficacy of rituximab for complicated SDNS. In this report, rituximab treatment of a child with both SDNS and idiopathic thrombocytopenic purpura resulted in the long-term remission of both diseases [30]. In addition, several case reports, case series, and survey studies found that rituximab treatment enabled most patients with complicated FRNS/SDNS to discontinue or reduce steroids and/or immunosuppressive drugs without relapse [21, 26, 27, 31–33]. Moreover, when one to five rituximab courses were given to 46 children with idiopathic nephrotic syndrome remission was maintained with steroids and calcineurin inhibitors. They were therefore diagnosed with complicated FRNS/SDNS, resulting in a 6-month probability of remission of 48 % after the first remission [34]. A multicenter off–on trial which primarily evaluated the effects of one or two doses of rituximab followed by withdrawal of immunosuppression on disease recurrence in 10 children and 20 adults with complicated FRNS/SDNS found that all patients were in remission after 1 year [35]. Furthermore, an open-label, randomized, controlled trial showed that rituximab plus lower doses of prednisone and calcineurin inhibitors were noninferior to standard doses of these agents in maintaining short-term remission in children with steroid- and calcineurin inhibitor-dependent nephrotic syndrome (i.e., complicated FRNS/SDNS) [36]. Collectively, therefore, these findings indicate that rituximab may be effective for children with complicated FRNS/SDNS. Kemper et al. performed a retrospective analysis of 37 patients with complicated SDNS who were treated with rituximab (375 mg/m^2^ given weekly for one to four courses). Time to first relapse was significantly shorter in patients receiving one to two courses compared with three to four initial infusions, whereas the proportion of patients with long-term remission was not related to the number of initial rituximab applications [37]. Kamei et al. retrospectively analyzed the risk factors for relapse in complicated SDNS treated with rituximab and found that only a history of SRNS was a statistically significant risk factor, whereas no other factor, including histologic findings (FSGS vs. minimal change nephrotic syndrome) was a significant risk factor [38]. Sinha et al. also reported that the period of remission in patients with a history of steroid resistance was significantly shorter than that in patients without such history [29].

Several papers reported that most patients were likely to relapse with B-cell recovery [32, 33, 39]. On the other hand, Shinha et al. found that the occurrence of relapse within 12 months of rituximab therapy was not associated with B-cell recovery at 4, 6, 8 or 12 months [29]. Colucci et al. recently reported that only delayed reconstitution of switched memory B cells, independent of immunosuppressive treatment, was protective against relapse after rituximab therapy [40].

Recently, Ravani et al. conducted a multicenter, open-label, noninferiority, randomized controlled trial to determine whether rituximab would be noninferior to steroids in maintaining complete disease remission in (not complicated) SDNS in children, and showed that rituximab was noninferior to steroids for the treatment of childhood SDNS [41].

The results of major case series, retrospective cohort studies and clinical trials, including our recent work [11] and a multicenter, open-label, randomized controlled trial recently carried out in Korea [42], of rituximab for complicated FRNS/SDNS are summarized in Table 2.

**Table 2:** Case series, retrospective cohort studies and clinical trials of rituximab for complicated FRNS/SDNS

Author/year [references]	Study design (no. of patients)	Rituximab dose	Major outcome
Guigonis/2008 [32]	Case series (*n* = 22)	2–4	Seven patients were nephrotic at the time of rituximab treatment, and remission was induced in three of them. One or more immunosuppressive treatments could be withdrawn in 19 (85 %) patients, with no relapse of proteinuria and without increasing other immunosuppressive drugs. Rituximab was effective in all patients when administered during proteinuria-free period in association with other immunosuppressive drugs. Adverse effects were observed in 45 % of cases, but most of them were mild and transient
Kamei/2009 [33]	Case series (*n* = 12)	1	All patients were able to discontinue steroids at a median of 74 days after treatment. The frequency of relapses per 6 months was significantly reduced (mean 2.83 vs. 1.08) and steroid-free period per 6 months was significantly increased (mean 7.0 vs. 68.0 days). Nine patients relapsed during the study period at a median of 129 days after treatment. None of the patients developed life-threatening adverse events
Gulati/2010 [34]	Case series (*n* = 24)	2	Twelve months after rituximab therapy, remission was sustained in 20 (83.3 %) patients. The mean number of relapses was significantly reduced (4.0 vs. 0.2 episodes/patient per year). The mean time to first relapse was 11.2 months. One or more immunosuppressive agents were withdrawn in 12 patients. One patient developed mild infusion reaction. None of the patients had serious infection or adverse event on follow-up
Ravani/2011 [37]	Multicenter, open-label, noninferiority randomized (1:1) controlled trial (*n* = 54)	1–2	Three-month proteinuria was 70 % lower in the rituximab arm as compared with standard therapy arm. The relapse rate in the rituximab arm was significantly lower than that in standard arm (18.5 vs. 48.1 %). Probabilities of being drug-free at 3 months were significantly higher in the rituximab arm (62.9 vs 3.7 %). Fifty percent of patients in the rituximab arm were in stable remission without drugs after 9 months. One patient developed bronchospasm and hypotension at the second rituximab infusion. Treatment was discontinued with spontaneous recovery. Two other cases required rituximab infusion in intensive care for initial bronchospasm, which improved after slowing the infusion rate
Kemper/2012 [38]	Retrospective cohort study (*n* = 37)	1–4	Twenty-six (70.3 %) patients remained in remission after 12 months. Time to first relapse was significantly shorter in patients receiving one or two compared to three or four initial infusion. However, the proportion of patients with long-term remission was not related to the number of initial rituximab applications
Ravani/2013 [35]	Single-arm clinical trial (*n* = 46)	1–5	Six-month probabilities of remission were 48 % after the first infusion and 37 % after subsequent infusions. 1- and 2-year remission probabilities were 20 and 10 %, respectively. The time to reconstitution of CD20 cells correlated with the duration of remission. Five patients required rituximab infusion in intensive care for initial bronchospasm, which improved after slowing the infusion rate. Two patients had neutropenia associated with transient viral infection
Iijima/2014 [11]	Multicenter, double-blind, randomized (1:1), placebo-controlled trial (*n* = 48)	4	The median relapse-free period was significantly longer in the rituximab arm than in the placebo arm (267 vs. 101 days). The relapse rate was significantly lower in the rituximab arm (1.54 vs. 4.17 relapses per person-year). Ten (42 %) patients in the rituximab arm and six (25 %) in the placebo arm had at least one serious adverse event, but the difference was not statistically significant
Ahn/2014 [43] (abstract)	Multicenter, open-label, randomized (2:1) controlled trial	1	At 6 months after treatment, the remission rates were 77. 1 % in the rituximab arm (*n* = 35) and 38.9 % in standard therapy arm (*n* = 18). Twenty-four (44.4 %) patients experienced mild and transient infusion reaction during rituximab infusion. However, no serious side effect was observed
Sinha/2015 [29]	Retrospective cohort study (steroid-dependent: *n* = 101, calcineurin inhibitor-dependent, steroid-resistant: *n* = 34)	2–4	In patients with steroid-dependent nephrotic syndrome, the mean relapse rate during 6 months after rituximab treatment was significantly lower than that before treatment (2.1 vs. 0.09). Also, in patients with calcineurin inhibitor-dependent, steroid-resistant nephrotic syndrome, the mean relapse rate during 6 months after rituximab treatment was significantly lower than that before treatment (2.0 vs. 0.2). Remission was longer in patients with steroid-dependent nephrotic syndrome compared with calcineurin inhibitor-dependent, steroid-resistant nephrotic syndrome (median 16 vs. 10 months)

## A multicenter, double-blind, randomized, placebo-controlled trial of rituximab therapy for childhood-onset complicated FRNS/SDNS [11]

The above-mentioned studies were case reports, case series, retrospective surveys, and single-arm, noninferiority or open-label trials, indicating the need for well-designed, randomized, controlled trials to determine the efficacy and safety of rituximab for children with complicated FRNS/SDNS. From 2008 to 2011, the Research Group of Childhood-onset Refractory Nephrotic Syndrome (RCRNS) in Japan conducted a multicenter, double-blind, randomized, placebo-controlled trial, RCRNS01 (Clinical Trials Registry ID: UMIN000001405), to evaluate the efficacy and safety of rituximab in childhood-onset complicated FRNS/SDNS. Simultaneously, a pharmacokinetic study of rituximab, RCRNS02 (Clinical Trials Registry ID: UMIN000001406), was performed. These two studies were investigator-initiated clinical trials to gain approval from the Ministry of Health, Labour and Welfare of Japan for rituximab treatment of patients with childhood-onset complicated FRNS/SDNS.

**Fig. 2:**
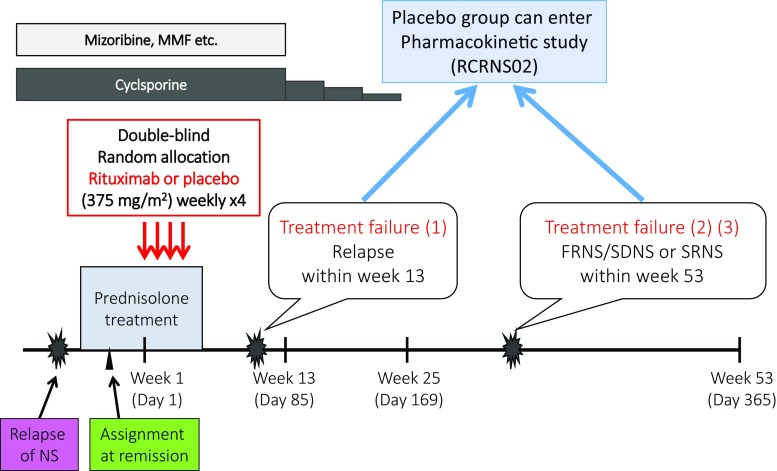
Experimental intervention in the RCRNS01 trial. *NS* nephrotic syndrome, *MMF* mycophenolate mofetil. Treatment failure, defined as *1* relapse by day 85, *2* diagnosis of FRNS or SDNS between day 86 and day 365, *3* diagnosis of steroid resistance during the observation period

In these studies, patients who had a relapse of nephrotic syndrome were treated with protocol-defined prednisolone therapy and underwent screening examinations. Investigators and patients were blinded to peripheral B cell counts, which were centrally monitored. Once patient eligibility, including steroid sensitivity, was verified, patients were randomly assigned (1:1) to two treatment groups. The patients, patients’ guardians, caregivers, treating physicians and individuals assessing outcomes were blinded to assignments. The rituximab group received 375 mg/m^2^ body surface area of intravenous rituximab (maximum 500 mg) once weekly for 4 weeks. The placebo group received placebo at the same frequency. After remission was achieved, prednisolone treatment was tapered gradually. On day 85, tapering of the CyA dose was started, and the drug was discontinued by day 169. Other immunosuppressive agents were discontinued by day 85. Patients who relapsed during the study period (1 year of follow-up) were treated with protocol-based prednisolone. Treatment failure was defined as follows: (1) relapse by day 85, (2) a diagnosis of FRNS or SDNS between day 86 and day 365, or (3) a diagnosis of steroid resistance during the observation period. If a patient showed treatment failure, the allocation code was disclosed. Those who had been randomized to the placebo group were able to enter a separately conducted rituximab pharmacokinetic study after discontinuation or completion of this trial (Fig. 2).

The primary endpoint was the relapse-free period, defined as the time of randomization to the time of the first relapse after the start of the study treatment. The secondary endpoints were time-to-treatment failure, relapse rate, time to FRNS or SDNS, and prednisolone dose after randomization. Adverse events including infection were also evaluated.

A total of 63 patients were screened, and 52 were randomized, 27 to the rituximab group and 25 to the placebo group. Twenty-four patients in each group (total 48) received the intervention and were included in the intention-to-treat analysis. Four patients in the rituximab group and 20 in the placebo group discontinued the intervention, mostly because of treatment failure. However, no patient dropped out of the study before the first relapse (the primary endpoint). All of the patients in the placebo group who experienced treatment failure were enrolled in RCRNS02.

**Fig. 3:**
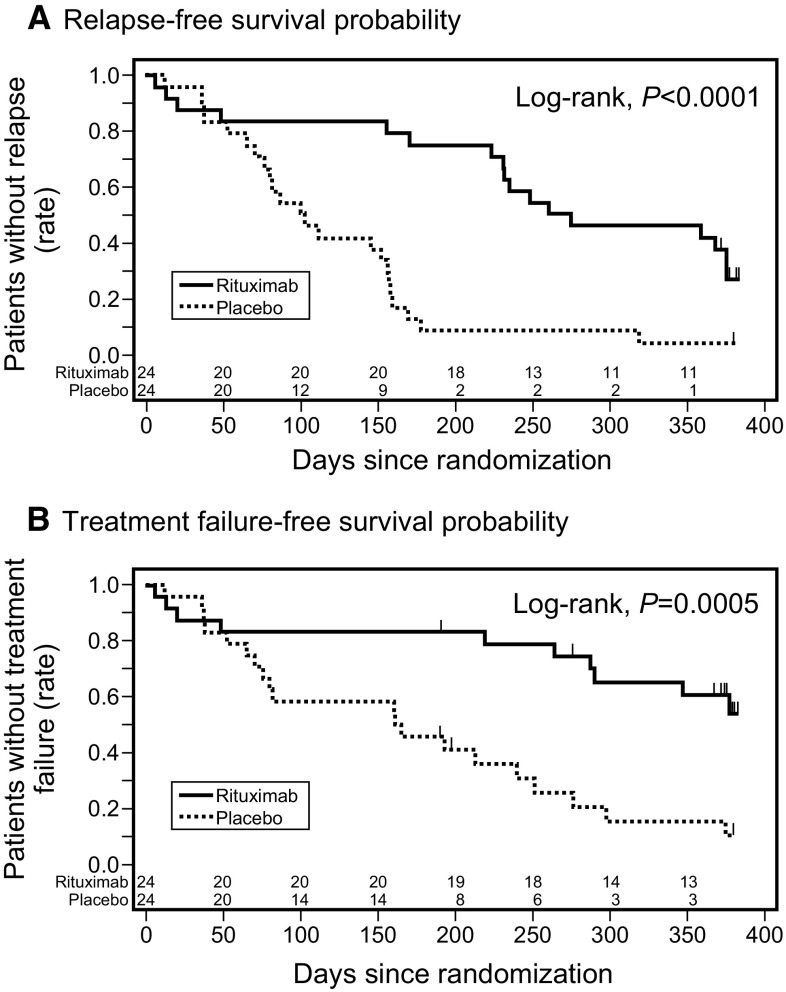
Kaplan–Meier analysis of **a** relapse-free survival and **b** treatment failure-free survival in the rituximab and placebo groups

Baseline characteristics were similar in the rituximab and placebo groups. The 50 % relapse-free period [267 vs. 101 days; hazard ratio (HR) 0.267, 95 % CI 0.135–0.528, *p* < 0.0001] (Fig. 3a) and the time-to-treatment failure (HR = 0.268, 95 % CI 0.122–0.589, *p* = 0.0005) (Fig. 3b) were significantly longer in the rituximab than in the placebo group. The relapse rate was significantly lower in the rituximab than in the placebo group [1.542 (29/18.81) vs. 4.171 (46/11.03) per person-years, HR = 0.370, 95 % CI 0.231–0.591, *p* < 0.0001], and the time to FRNS or SDNS was significantly longer in the rituximab than in the placebo group (HR = 0.169, 95 % CI 0.061–0.464, *p* = 0.0001). Daily steroid dose after randomization was significantly lower in the rituximab than in the placebo group (9.12 ± 5.88 vs. 20.85 ± 9.28 mg/m^2^/day, *p* < 0.0001). In this trial, no deaths were reported and the majority of adverse events were mild. Rates of serious adverse events [42 % (10/24) vs. 25 % (6/24), Fisher’s exact test, *p* = 0.3587] and infusion reaction [79 % (19/24) vs. 54 % (13/24), Fisher’s exact test, *p* = 0.1246] were similar in the two groups, and no patient in either group experienced Grade 3 or 4 infusion reactions. These findings indicated that rituximab was safe and effective, at least for 1 year, in the treatment of childhood-onset, complicated FRNS/SDNS.

Based on these results, the Ministry of Health, Labour and Welfare (MHLW) of Japan approved the use of rituximab for patients with complicated FRNS/SDNS on August 29, 2014. An early postmarketing phase vigilance was conducted in Japan from August 29, 2014 to February 28, 2015, and collected reports of 26 side effects from 19 patients including adults. Reports of serious adverse events among pediatric patients were limited to two patients. The serious adverse event of one male patient <10 years was rash, dyspnea, wheezing, and vomiting. All symptoms appeared within the day of administration and relieved. There was no report on concomitant drug or other complications. Another was a female patient <10 years, and granulocytopenia was reported at 81 days, but she recovered. She had also been receiving cyclosporine and prednisolone [43].

## Adverse effects of rituximab

Rituximab is safe and well tolerated in most patients. However, rituximab has been associated with several serious adverse events, including fatal hepatitis induced by rituximab reactivation of hepatitis B virus [44] and progressive multifocal leukoencephalopathy [45]. Rituximab has been associated with serious adverse events in children with complicated FRNS/SDNS, including pulmonary fibrosis [46], fulminant myocarditis [47], pneumocystis pneumonia, [32] immune-mediated ulcerative colitis [48] and agranulocytosis [49]. Recently, two patients developed hypersensitivity reactions, including anti-rituximab antibodies, during a second course of rituximab infusion [50]. The 3-year mortality rate following the initiation of anti-CD20 therapy in patients with various autoimmune diseases was reported to be 3 %, with most deaths due to infection [51]. Prospective cohort studies are needed to determine the long-term consequences of rituximab therapy in children with complicated FRNS/SDNS and SRNS.

After the end-August 2014 approval of rituximab for complicated FRNS/SDNS by MHLW, Zenyaku Kogyo Co., Ltd. (Tokyo, Japan) and Chugai Pharmaceutical Co., Ltd. (Tokyo, Japan) initiated “all-case” drug use-results survey in Japan to confirm the safety and efficacy of rituximab administered to complicated FRNS/SDNS patients in clinical practice [52]. Observational period of the survey is 2 years, and as of April 15, 2016, 911 patients including 413 patients under 15 years were enrolled to the survey. The re-evaluation results of the safety and efficacy form large number of patients will be obtained in near future.

## Conclusions and future perspectives

Rituximab is a promising treatment for complicated FRNS/SDNS in children. However, this drug does not cure the disease, as all patients treated with rituximab in the RNRNS01 trial had relapsed by 19 months after randomization [11]. Further modifications of rituximab treatment, including adjunct immunosuppressive therapies and repeated courses of rituximab, may be necessary to extend the relapse-free period. Repeated rituximab administrations just after the re-emergence of B cells or 3-month interval were reported to be effective for complicated FRNS/SDNS [53, 54]. However, the effect of persistent B cell depletion on the developing immune system in children is unknown. Also, the lack of efficacy of vaccination under persistent B cell depletion is a critical problem in children. Ito et al. reported that maintenance therapy with mycophenolate mofetil after rituximab treatment was effective in children with complicated FRNS/SDNS [55]. A multicenter, double-blind, randomized, placebo-controlled trial, JSKDC07 (Clinical Trials Registry ID: UMIN000014347), assessing the efficacy and safety of mycophenolate mofetil after rituximab therapy in children with complicated FRNS/SDNS was started in 2015 in Japan. The primary endpoint of JSKDC07 is the time-to-treatment failure (development of frequent relapses, steroid dependence or steroid resistance). Further studies are needed comparing the efficacy, safety, and cost-effectiveness of various rituximab-dosing regimens and B cell-driven regimens in children with complicated FRNS/SDNS. At this time, there is no evidence that rituximab is effective in patients with refractory SRNS. However, most children with refractory SRNS achieved remission by following treatment with rituximab combined with conventional methylprednisolone pulse therapy and immunosuppressive agents [12]. A multicenter, single-arm trial assessing the efficacy and safety of rituximab combined with methylprednisolone pulse therapy and immunosuppressive agents for refractory SRNS in children, JSKDC08 (Clinical Trials Registry ID: UMIN000014895), will be started in 2016 in Japan. The primary endpoint of JSKDC08 is the rate of complete remission at 6 months after the start of the test treatment.

Scientists have been developing new reagents to attain higher treatment efficiencies and to achieve increased benefit over rituximab. There are now several anti-CD20 monoclonal antibodies with FDA approval, including ofatumumab and obinutuzumab. Basu and Bonanni et al. reported that ofatumumab may be an effective treatment in managing rituximab-resistant refractory SRNS [56, 57]. Further studies are needed to examine the efficacy and safety of new anti-CD20 monoclonal antibodies for the treatment of difficult-to-treat nephrotic syndrome.
